# Applications of a Semi-Crystalline Thermoplastic Constitutive Model to Mechanical Responses of Electronic Connector Structures

**DOI:** 10.3390/ma14195812

**Published:** 2021-10-04

**Authors:** Ting-Chuan Huang, Kuo-Chi Liao

**Affiliations:** Department of Biomechatronics Engineering, National Taiwan University, No. 1, Sec. 4, Roosevelt Road, Taipei 10617, Taiwan; D08631002@ntu.edu.tw

**Keywords:** constitutive model of thermoplastic semi-crystalline polymers, failure assessment, finite element analysis, retention force, electronic connector

## Abstract

The retention force of electronic connectors, in general one of the essential specification requirements, is defined as a maximum force of metallic terminals withdrawn out of the corresponding plastic housing. Accurate prediction of the retention force is an important issue in the connector design stage; however, it is not an easy task to accurately assess the retention force based on the authors’ knowledge. A finite element analysis is performed in conjunction with a self-coded user subroutine accounting for relaxation/creep behaviors of semi-crystalline thermoplastic polymers under various loading conditions in order to appraise the mechanical performance of the plastic base structure. Material parameters adopted in the constitutive model are evaluated by utilizing the automated design exploration and optimization commercial software. Applications of the developed subroutine with several damage criteria to assess retention forces of two electronic connectors were conducted. Retention forces predicted by utilizing the current constitutive model agreed fairly well with the associated experimental measurements. A dramatic improvement of the underestimation of the retention force based on the approach commonly adopted in the industry is also demonstrated here.

## 1. Introduction

Thermoplastic polymers are broadly used as engineering materials in modern industry, from automotive systems to electronic devices. An electronic connector, used for electrical signal transmission, is basically comprised of two major components, these being metallic terminals and a polymeric housing. Terminals are secured into the housing via the interference of barbs designed in the terminal and the polymeric structure by using a fixture. The retention force of the connector, defined as the peak force of the terminal withdrawn out of the associated housing, is required to be sufficient to prevent embedded terminals from being detached from the housing under relatively critical conditions such as shock and vibration. Accurate prediction of the retention force is thus an important issue, especially in the connector design stage. Nonetheless, it is generally known that the retention force is not easy to be appropriately estimated due to the complex mechanical responses and fracture mechanisms of thermoplastic polymers.

In order to properly illustrate the dependence of time, strain rate, and temperature on stress-strain behaviors of thermoplastic polymers, various constitutive models were developed based on experimental observations. Colak [1] modified the viscoplasticity theory based on overstress (VBO) to describe the nonlinear strain rate sensitivity, multiple creep, and recovery behaviors of amorphous polymer polyphenylene oxide (PPO). Two tensor-based state variables, the equilibrium and kinematic stresses and two scalar-based state variables, the drag and isotropic stresses, were incorporated into this unified model. Several conditions, including uniaxial strain-controlled loading and unloading with various strain rates, multiple creep at room temperature, and recovery at zero stress were numerically investigated and validated by the corresponding experimental date presented in the literature. Dusunceli and Colak [2] extended the modified VBO model to account for the crystallinity ratio on mechanical responses of semi-crystalline polymeric materials such as ultra-high molecular weight density polyethylene (UHMWPE). They concluded that the further modified VBO model in which amorphous and crystalline phases in parallel is able to rationally reproduce the examined material behaviors. Ayoub et al. [3] developed a physically-based inelastic model under finite strain formulation to describe mechanical responses of high density polyethylene (HDPE). The visco-hyperelastic network resistance was arranged in parallel with the viscoelastic–viscoplastic intermolecular resistance, where the amorphous and crystalline phases are explicitly taken into consideration. Numerical results of HDPE associated with monotonic loading/unloading, stress relaxation, and cyclic loading behaviors at different strain levels were reported and experimentally verified. Effects of the crystallinity on the loading and unloading behaviors were also explored. Recently, Barba et al. [4] stated that the level of reorientation of crystalline and amorphous phases of polyether-ether-ketone (PEEK) is strongly influenced by variables such as temperature and strain rate. They thus proposed a constitutive model accounting for effects of these mechanisms on mechanical behaviors of such polymers within different thermal regions. Deformation mechanisms of UHMWPE at high strain rates and large strain are quite different from those involved in classical semi-crystalline polymers, and the reason for this phenomenon could be that chain disentanglements are almost impossible for very long macromolecules, even at temperatures far above the melting point. Bernard et al. [5] then developed a three-dimensional model to predict the mechanical behavior of UHMWPE, while the evolution of microstructures between crystalline lamellae and the confined amorphous phase during the deformation process can also be taken into account. Numerical predictions are in fair agreement with the associated experimental results for the specimen under both loading and unloading conditions. Xu et al. [6] modified the existed constitutive model with the Drucker Prager yield criterion to assess mechanical responses of Polyamide 66 (PA66) subjected to rather high-rate loading conditions. Serval sets of Taylor impact tests for PA66 bars were performed to verify the accuracy of the three-dimensional constitutive model, and the deformed shapes of the specimen were found to be similar to these obtained from the tests.

Gearing and Anand [7] used the maximum principal stress damage criterion to determine whether the polymer fracture is induced by the craze flow. Krairi and Doghri [8] developed a progressive ductile damage criterion in the view of a framework of thermodynamics. Stiffness of the thermoplastic polymer is regarded as a function of the damage parameter. Progressive damage of the material, up to rupture, under various loading conditions was validated by the corresponding measurements. Torres et al. [9] investigated the failure of HDPE subjected to the dynamic impact. The maximum principal logarithmic strain was taken as a criterion to successfully appraise the pattern of fracture. Recently, Fedulov et al. [10] characterized the damage accumulation of the polymer in dependence on the stress state type.

Academic studies related to applications of the advanced constitutive model suitable for the thermoplastic materials to industrial products under practical operation conditions are not broadly reported, and there are even fewer research articles focusing on the assessment of the retention force of electronic connectors. The constitutive model proposed by Ayoub et al. [3], with various damage criteria that are self-coded as a subroutine implemented into the widely-used finite element analysis commercial package ABAQUS [11] for capturing mechanical responses of semi-crystalline thermoplastic polymers in the current study. Retention forces based on the simulations are further validated by those based on the corresponding experimental measurements.

## 2. Materials and Methods

### 2.1. Experiments

In order to examine the rate-dependent mechanical response of semi-crystalline polymers, a series of uniaxial compression tests for both polyamide 4T (PA4T) and liquid crystalline polymer (LCP) were conducted at different strain rates of 0.001 $s^{-1}$ and 0.1 $s^{-1}$. Cylinder specimens with a diameter of 12.7 mm and a height of 25.4 mm according to the American Society for Testing and Materials (ASTM) D695 standard were prepared. Reaction forces and displacements of the compressive head were monitored until the specimen was ruptured (with five repetitions).

**Figure 1:**
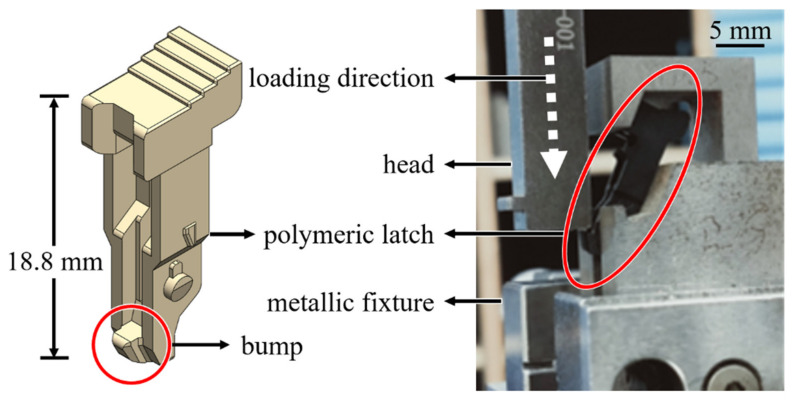
An experimental set-up for the measurement of the strength of the latch of DDR4 connector.

To further assess the appropriateness of the choice of damage criteria presented later, the strength of an injection-molded PA4T latch of DDR4 connector under multiaxial loading conditions was conducted, as shown in Figure 1. The polymeric latch was installed into a metallic fixture, while a head attached to a load cell was moved downwards with a constant speed of 0.1 mm/s to compress against the bump until the latch rupture occurred. Associated reaction forces and displacements of the compressive head were then recorded (with five repetitions).

**Figure 2:**
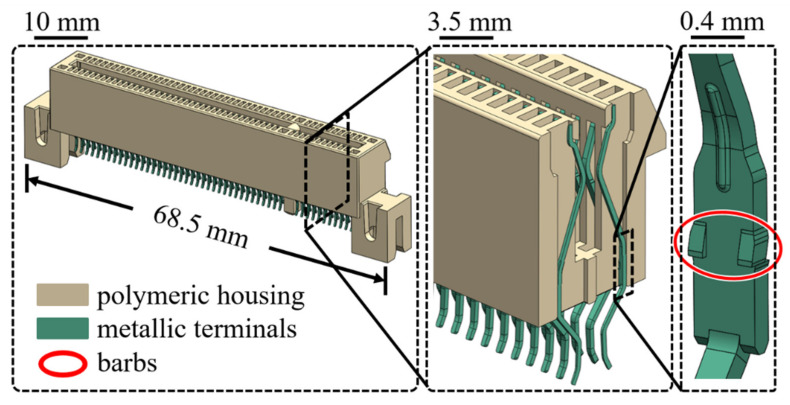
A drawing of PCIE connector.

**Figure 3:**
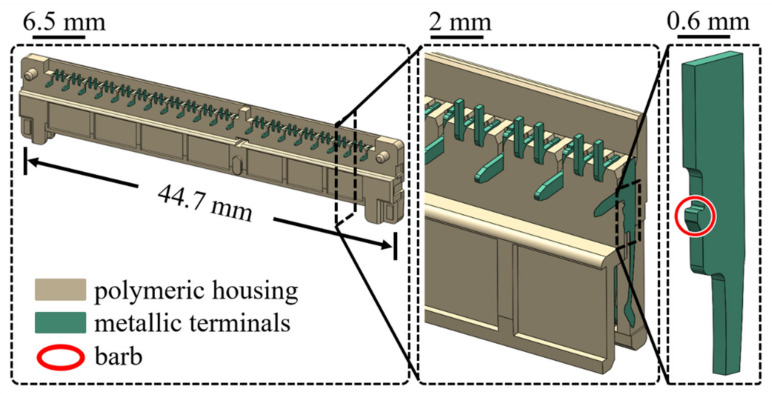
A drawing of SSD connector.

**Figure 4:**
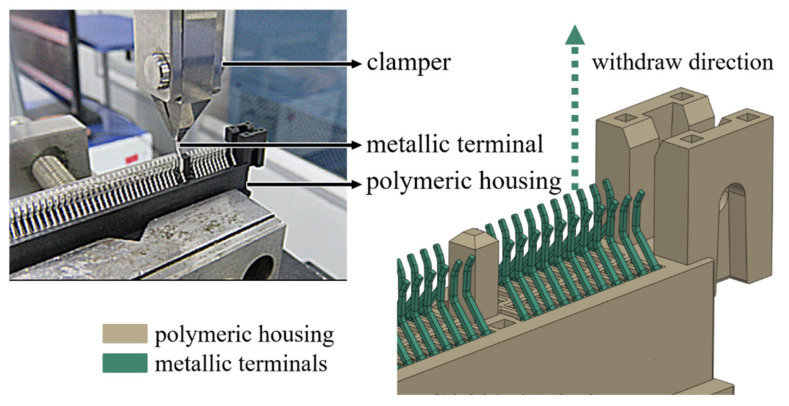
A schematic diagram of experimental set-up for the measurement of the retention force.

**Figure 5:**
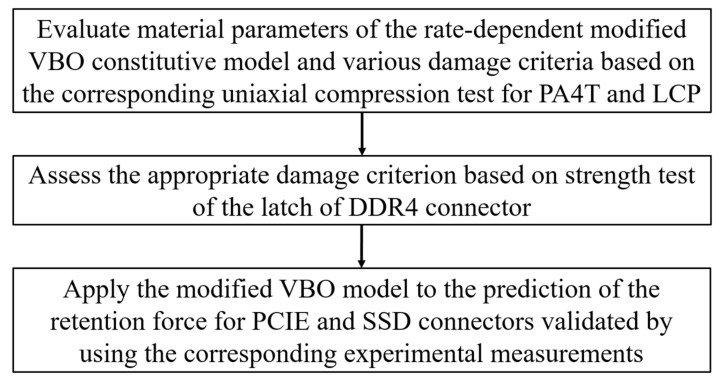
A flowchart of the logical relationships among three experiments.

Two types of the electronic connector, PCIE (with a PA4T housing) and SSD (with a LCP housing) as shown in Figure 2 and Figure 3, respectively, were chosen for the investigation of the retention force here. A schematic diagram of experimental set-up for measurements of the retention force is displayed in Figure 4. The terminal gripped by a clamper was withdrawn from the polymeric housing at a constant velocity of 0.83 mm/s, which was numerically converted into the resulting representative strain rate level of one-tenth per second. Two strain rate levels (0.001 and 0.1 1/s) applied to the cylindrical specimen under the uniaxial compression test mentioned above thus reasonably correlate to the current experimentally investigated strain rate regime. Corresponding forces detected by a load cell attached to the clamper were then recorded (with five repetitions). A flowchart providing logical relationships among the above three experiments is shown in Figure 5.

### 2.2. Simulations

**Figure 6:**
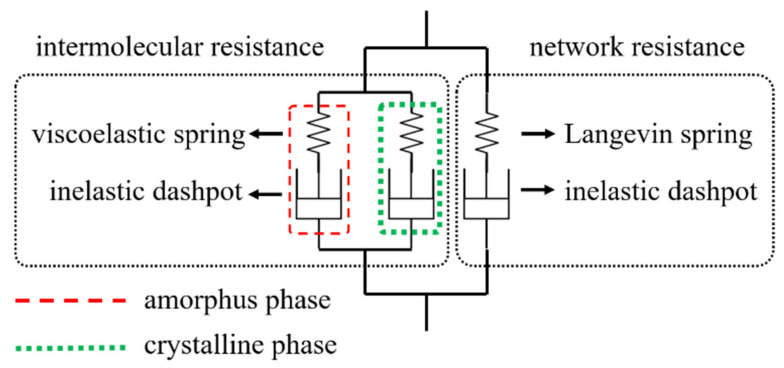
Schematic representation of the modified VBO model.

The constitutive model proposed by Ayoub et al. [3] is chosen here to describe mechanical responses of the polymeric material. A representation of the model consisting of the intermolecular resistance A (a nonlinear viscous spring in series with a viscous dashpot) and network resistance B (a Langevin spring in series with a viscous dashpot) arranged in parallel is schematically illustrated in Figure 6. Furthermore, two different deformation mechanisms in intermolecular resistance take place due to the presence of amorphous and crystalline phases in the semi-crystalline polymer. The coupling between both deformation modes in the intermolecular resistance can be evaluated via a composite framework. It is assumed that the amorphous intermolecular resistance acts in parallel with the crystalline intermolecular resistance. Due to the attributes of the arrangement, the deformation gradient of each resistant part equals the total deformation gradient F while the overall Cauchy stress T can be regarded as the tensorial sum of each resistance part.

(1) $$F=F_{A}=F_{B},$$

(2) $$T=T_{A}+T_{B},$$

Deformation gradients of both intermolecular resistance and network resistance can be respectively decomposed into elastic and inelastic parts in a multiplicative manner.

(3) $$F_{A}=F_{A}^{i}=F_{A}^{e\_i}F_{A}^{p\_i},$$

(4) $$F_{B}=F_{B}^{e}F_{B}^{p},$$

in which the variables with superscript ‘i’ represent either amorphous phase ‘a’ or crystalline phases ‘c’ for simplification. The Cauchy stress of the intermolecular part is determined by using the rule of mixture.

(5) $$T_{A}=\chi_{c}T_{A}^{c}+\left(1-\chi_{c}\right)T_{A}^{a},$$

in which $\chi_{c}$ is the volume fraction of the crystalline phase. The Cauchy stress of each phase in the intermolecular resistance is related to the corresponding elastic deformation gradient by the following constitutive law for a linear elastic spring.

(6) $$T_{A}^{i}=\frac{1}{J_{A}^{i}}C_{A}^{e_{i}}ln\left(V_{A}^{e_{i}}\right),$$

(7) $${\left(C_{A}^{e\_i}\right)}_{klmn}=\frac{\varphi_{A}^{i}E_{A}^{i}}{2\left(1+\nu_{A}^{i}\right)}[\left(\delta_{km}\delta_{ln}+\delta_{kn}\delta_{lm}\right)+\frac{2\nu_{A}^{i}}{1-2\nu_{A}^{i}}\delta_{kl}\delta_{mn}],$$

here $J_{A}^{i}$ is the volumetric change, $ln\left(V_{A}^{e\_i}\right)$ is defined as the Hencky strain, $\nu_{A}^{i}$ is Poisson’s ratio, and $E_{A}^{i}$ is Young’s modulus. Kronecker delta $\delta_{ab}$ equals 1 when a=b and 0 when a≠b. The modified viscoplasticity theory based on overstress is introduced in the stress-strain relationship of the intermolecular resistance, via the implementation of Equations (8) and (9) to obtain the change in the elastic stiffness of the polymeric materials under loading/unloading conditions.

(8) $$\varphi_{A}^{i}=1-\beta^{i}{\left(\frac{\eta^{i}}{r^{i}}\right)}^{\alpha^{i}},$$

(9) $$\eta^{i}={\left[\left(G^{\prime \_i}-K^{\prime \_i}\right):\left(G^{\prime \_i}-K^{\prime \_i}\right)/2\right]}^{1/2},$$

where $\beta^{i},\;\alpha^{i},$ and $r^{i}$ are material constants. $G^{\prime \_i}$ and $K^{\prime \_i}$ are respectively the deviatoric form of two tensorial state variables, namely equilibrium stress G and kinematic stress K. The evolution of equilibrium and kinematic stresses, ${\dot{G}}^{\prime_{i}}$ and ${\dot{K}}^{\prime_{i}}$, are also expressed in deviatoric form.

(10) $${\dot{G}}^{\prime_{i}}=\Psi^{i}\frac{{\dot{T}}_{A}^{\prime \_i}}{E_{A}^{i}}+\Psi^{i}{\left(\sqrt{3}\frac{\tau_{A}^{i}}{H^{i}}\right)}^{m_{i}}(\frac{T_{A}^{\prime \_i}-G^{\prime \_i}}{\sqrt{3}\tau_{A}^{i}}-\frac{G^{\prime \_i}-K^{\prime \_i}}{r^{i}})+(1-\frac{\Psi^{i}}{E_{A}^{i}}){\dot{K}}^{\prime \_i},$$

(11) $${\dot{K}}^{\prime_{i}}=\frac{1}{\sqrt{2}}\frac{{\left(T_{A}^{\prime_{i}}\cdot T_{A}^{\prime_{i}}\right)}^{\frac{1}{2}}}{\sqrt{3}\tau_{A}^{i}+g^{i}}\frac{E_{A}^{i}E_{t}^{i}}{\left(E_{A}^{i}-E_{t}^{i}\right)}D_{A}^{p\_i},$$

in which $T_{A}^{\prime \_i}$ is the deviatoric form of Cauchy stress, $\tau_{A}^{i}$ is the effective shear stress, $H^{i}$ is the drag stress, $m_{i}$ is the viscosity coefficient, $g^{i}=(G^{\prime \_i}\cdot G^{\prime \_i}/2)1/2$ is the effective value of equilibrium stress, $E_{t}^{i}$ is the tangent modulus, and $\Psi^{i}$ is the shape function expressed as

(12) $$\Psi^{i}=\Psi_{1}^{i}+(\frac{c_{2}^{i}-\Psi_{1}^{i}}{\exp(c_{3}^{i}\gamma_{A}^{p\_i})}),$$

with

(13) $$\Psi_{1}^{i}=c_{1}^{i}+\left(1+c_{4}^{i}\left(\frac{g^{i}}{r^{i}+c_{5}^{i}{\left(\tau_{A}^{i}\right)}^{2}}\right)\right),$$

where $c_{1}^{i}\;\sim \;c_{5}^{i}$ are shape coefficients and $\gamma_{A}^{p\_i}$ is the inelastic shear strain rate.

Furthermore, the stress–strain behavior of the network resistance is given by the eight-chain rubber model (Arruda and Boyce [12]).

(14) $$T_{B}=\frac{1}{J_{B}}\frac{nk\theta }{3}\frac{\sqrt{N}}{{\bar{\lambda }}_{B}^{e}}L^{-1}(\frac{{\bar{\lambda }}_{B}^{e}}{\sqrt{N}})[{\bar{B}}^{e}-{\left({\bar{\lambda }}_{B}^{e}\right)}^{2}I],$$

where n is a material constant, k is the Boltzman’s constant, θ is absolute temperature, $J_{B}$ is the network volume change, ${\bar{B}}^{e}$ is the isochoric left Cauchy-Green tensor, ${\bar{\lambda }}_{B}^{e}$ is the stretch of chain, and $L^{-1}\left(x\right)=x\left(3-x^{2}\right)/\left(1-x^{2}\right)$ is the inverse of Langevin function. Note that the temperature dependence of relaxation can be taken into account in the network resistance. Detailed description of the present constitutive model can be found in Ayoub et al. [3].

**Figure 7:**
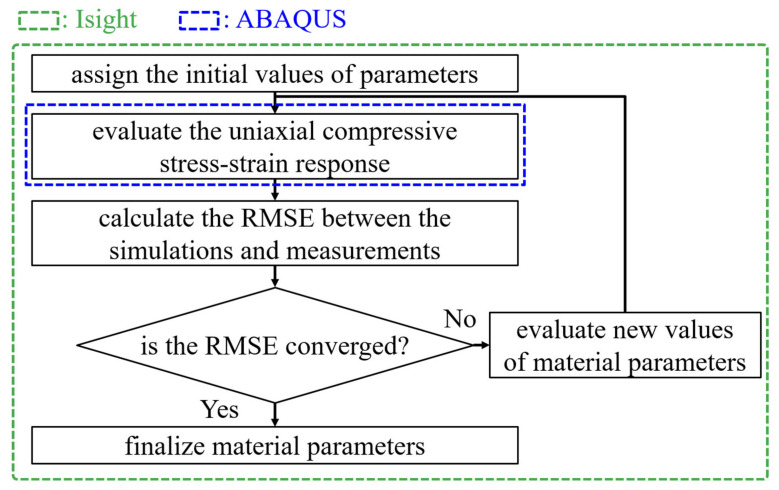
A flowchart of the evaluation of material parameters.

The users’ subroutine VUMAT, which accounts for the constitutive model mentioned above, is coded and the ABAQUS/Explicit [11] solver is adopted in the present study. In order to evaluate all material parameters (up to 29 constant values) embedded in the mathematical models for PA4T and LCP, the stress-strain relationships under the uniaxial compression condition at different strain rates based on the simulations are fitted with those based on the experiments. Automate design exploration and optimization software Isight [13] linking to ABAQUS [11] is used to adjust the stress-strain response. An analysis model based on the uniaxial compression test is constructed, and initial values of material parameters is imported into ABAQUS [11] to assess the stress-strain response of PA4T and LCP. Mesh sensitivity tests for all subsequent numerical simulations are performed. The root-mean-square error (RMSE) between the measurements and simulations is then estimated. The Hooke-Jeeves algorithm built in Isight [13], which is one of the popular choices among the local search algorithms, is adopted to minimize RMSE by adjusting values of these parameters, and new values are then introduced into ABAQUS [11] again. The above procedures will be repeated until RMSE is converged. A flowchart of the evaluation of material parameters is demonstrated in Figure 7.

Four developed damage criteria are examined to evaluate the failure of the polymeric materials. (a) Maximum principal strain criterion: A local region of the structure with maximum principal strain larger than a certain threshold $\varepsilon_{1,\;cr}$ is regarded as damage. (b) Equivalent viscoplastic strain criterion: A parameter $D^{vp}=\int \frac{d\varepsilon_{eq}^{vp}}{\varepsilon_{D}^{vp}}$ is defined to model the damage response, where $\varepsilon_{D}^{vp}$ is a dependency determined experimentally for the given material. The material stiffness is zeroed when $D^{vp}=1$. (c) Damage accumulation criterion: Material damage follows the Chaboche–Lemaitre evolution law $\left\{\begin{array}{c} \dot{D}=Y{\dot{\varepsilon }}_{eq}^{vp}>0,\;if\;\varepsilon^{vp}>p_{D}\\ D=D_{cr}\to \;crack\;initiation \end{array}\right.$, where Y is a material constant and the equivalent viscoplastic strain rate ${\dot{\varepsilon }}_{eq}^{vp}$ is defined as $\sqrt{\frac{2}{3}{\dot{\varepsilon }}^{vp}:{\dot{\varepsilon }}^{vp}}$ related to the viscoplastic strain rate ${\dot{\varepsilon }}^{vp}$. Here $p_{D}$ is a damage threshold, D is the accumulated damage, and $D_{cr}$ is a critical damage value. (d) Craze-initiation criterion: The craze-initiation may be taken to occur when the maximum principal stress $\sigma_{1}$ reaches a critical value $\sigma_{1,cr}\left(\sigma_{m}\right)=f_{1}+\frac{f_{2}}{\sigma_{m}}$, in which $f_{1}$ and $f_{2}$ are material constants and $\sigma_{m}$ is the mean normal stress.

**Figure 8:**
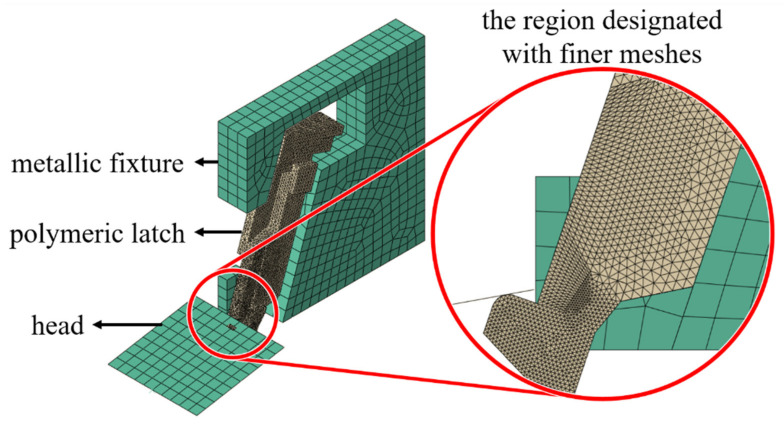
An analysis model of the latch of DDR4 connector.

Mechanical behaviors of the latch of DDR4 connector under the compression conditions as described in the previous section are numerically examined. Both the head and the metallic fixture are assumed to be rigid bodies since they are much stiffer than the polymeric latch. Four-node tetrahedron elements with reduced integration are assigned to the latch while a region enduring large deformation illustrated in an insert of Figure 8 is designated with finer meshes. The coefficient of friction of interfaces between the latch and the rigid surface was set 0.2. The head was moved vertically down until its travelling distance reached a specified magnitude.

**Figure 9:**
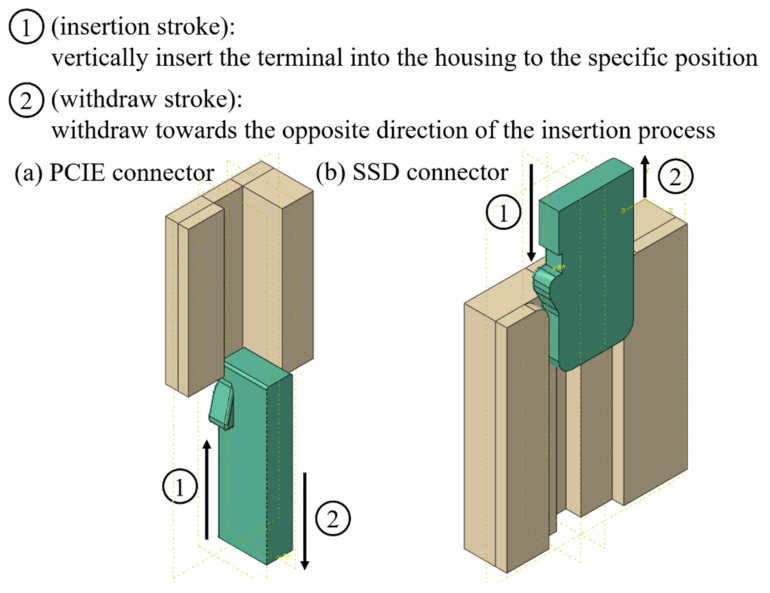
Simplified analysis models of PCIE and SSD connectors.

Geometry models of PCIE and SSD connectors are reasonably simplified in the numerical analysis as shown in Figure 9 for the significant reduction of computational cost. The metallic terminal can be regarded as a rigid body in the numerical analysis. Eight-node brick elements with reduced integration and discrete rigid elements are assigned to the housing and the terminal, respectively. The coefficient of friction of all interfaces between the housing and the terminal was also set 0.2. The terminal is first vertically inserted into the housing to the specific position (insertion stroke), and it is then withdrawn towards the opposite direction of the insertion process (withdraw stroke) at the same speed as that used in the experiments.

It should be noted that in the connector industry, based on the authors’ understanding, the polymeric housing is usually regarded as the simple isotropic elastic-plastic material for the retention force numerical analysis. Comparisons of the simulation results based on the modified VBO model and the isotropic elastic-plastic model will be presented in the next section.

## 3. Results and Discussion

**Figure 10:**
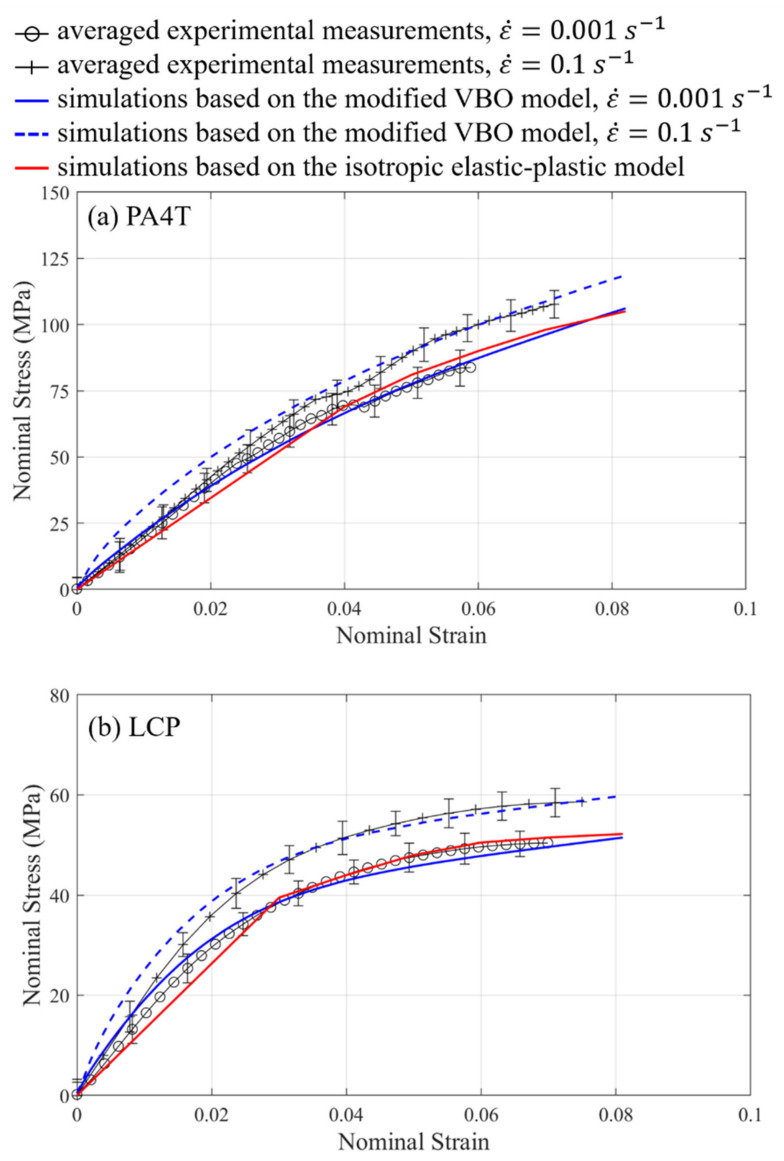
Stress-strain relationships of (**a**) PA4T (**b**) LCP under the uniaxial compression conditions based on the experiments and simulations.

**Figure 11:**
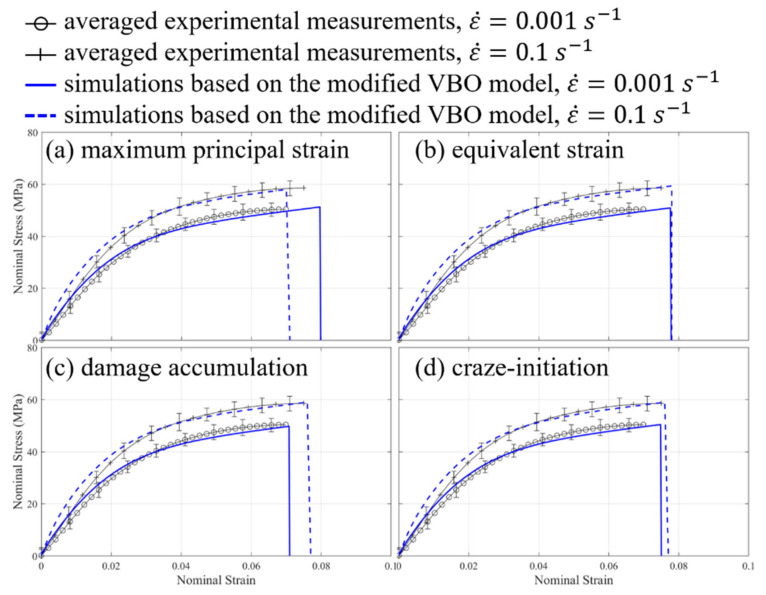
Comparisons of stress-stain relationships of LCP under the uniaxial compression conditions based on the simulations with various damage criteria.

Figure 10a,b show the stress-strain relationships of PA4T and LCP based on averaged experimental measurements and simulations under the uniaxial compression conditions. After implementing the optimization scheme into the numerical analysis, simulated stress-stain curves based on one appropriate set of material parameters listed in Table 1 are in good agreement with the measured ones. Material parameters used in the isotropic elastic-plastic model for PA4T/LCP are Young’s modulus of 1750/1300 MPa, Poisson’s ration of 0.4/0.4, and yield point of 70/40 MPa. Experimental measurements further demonstrate that both polymers with higher strain rates give rather stiffer responses. Simulation results also display that the modified VBO model can properly illustrate the strain rate effect, while the isotropic elastic-plastic model is incapable of accounting for the strain rate dependence of polymers. Moreover, the stress-strain relationships of LCP, for example, based on the simulations with the aforementioned four damage criteria under the uniaxial compression conditions are compared to those based on the experiments as shown in Figure 11. Table 2 lists the corresponding parameters adopted for various damage criteria. The predicted rupture strain based on lower strain rate conditions occurs earlier by using the maximum principal strain damage criterion, while the assessed rupture strains based on two distinct strain rates seems to be the same via the equivalent strain damage criterion. However, these estimations are counter to the experimental observations. On the other hand, both the damage accumulation and craze-initiation criteria in some sense can describe the phenomenon that higher strain rate induces larger rupture strain consistent with the corresponding measurements. Furthermore, the damage accumulation criterion gives good estimated rupture strains in comparison with the experiments. Applications of these four damage criteria to PA4T are also carried out (results not shown here), and trends of simulation results based on PA4T are similar to those based on LCP, as mentioned above.

**Figure 12:**
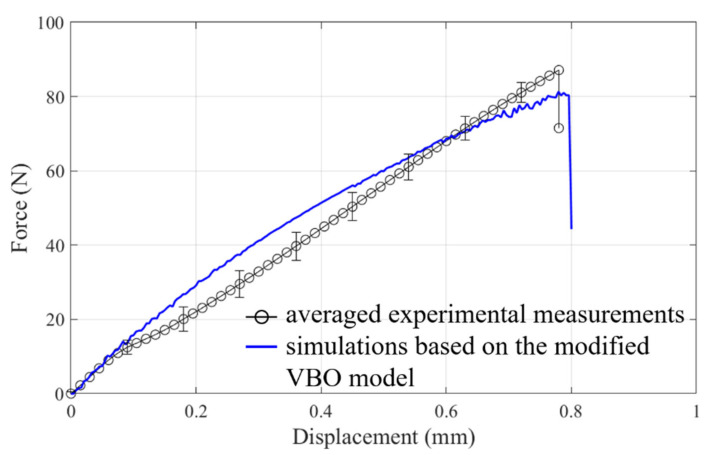
Force-displacement responses of the latch of DDR4 connector based on the experiments and simulations.

**Figure 13:**
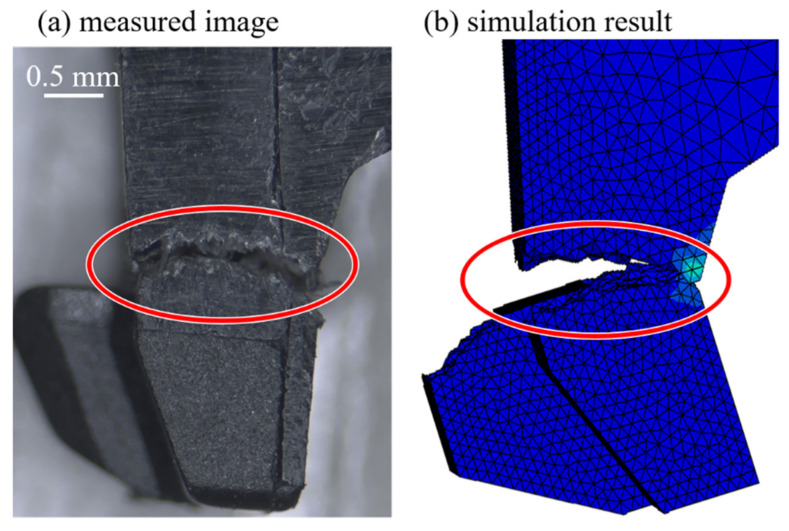
Comparisons of the rupture location based on the experiments and simulations.

Numerical simulations of the latch of the DDR4 connector under rather complicated loading conditions with four damage criteria were then performed. For the sake of concise presentation, only the force-displacement responses subjected to the head based on the calculations with the damage accumulation criterion are shown in Figure 12, and they are in fair agreement with the corresponding measurements, while the sudden drop of the force is due to the occurrence of the structure rupture. Deviations of the slope of the measured and simulated force-displacement relationships becomes moderately significant when the head displacement reaches approximately 0.11 mm. This phenomenon could be attributed to the slight slip occurring between the head and bump in the experiment. It should be noted that the element deletion technique is implemented into the numerical analysis to demonstrate the rupture of the structure. Figure 13 shows that the predicted rupture location is consistent with the experimental observation. Calculated strength investigations of the latch based on the other three damage criteria, however, cannot give fair predictions in comparison with the experiments.

**Figure 14:**
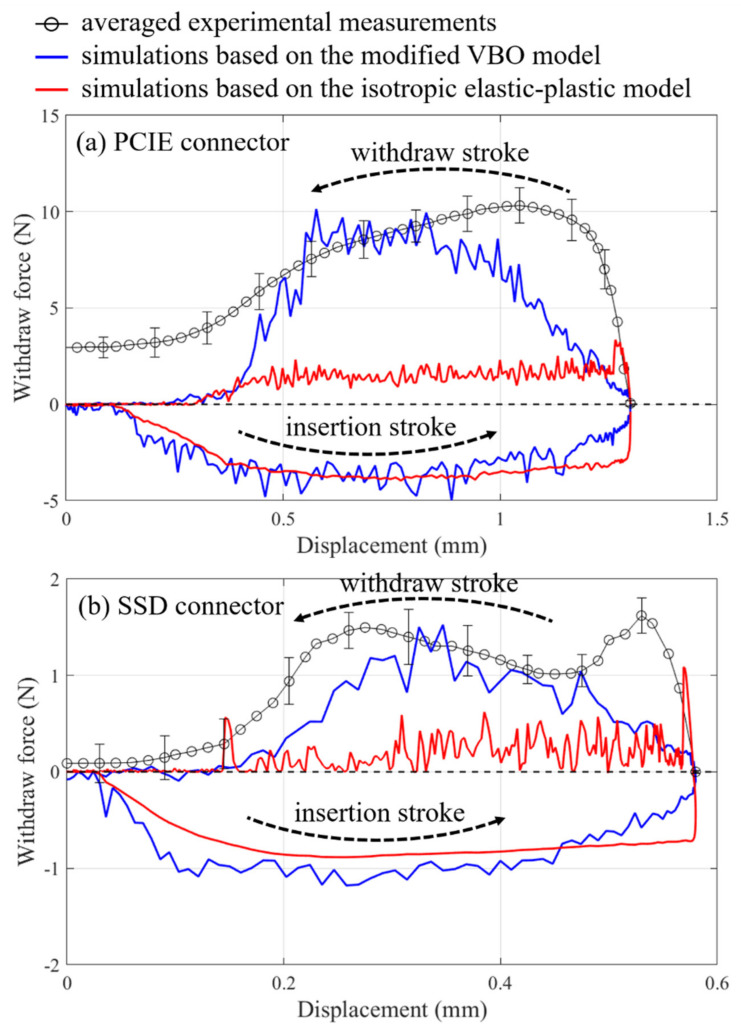
Force-displacement relationships of (**a**) PCIE connector and (**b**) SSD connector based on the experiments and simulations during the insertion/withdraw strokes.

Insertion/withdrawal force histories with respect to the displacement based on the simulations with the damage accumulation criterion of PCIE and SSD connectors are shown in Figure 14a,b, respectively. It can be remarked that the insertion forces based on the modified VBO model and the isotropic elastic-plastic model are rather similar. The zigzag phenomena of the simulated responses could be attributable to the numerical instability caused by the mechanism of progressive element deletion, especially along the withdraw stroke of the terminal. It should be noted that (a) various coefficients of friction are used in the numerical analysis; however, results based on the coefficient of friction of 0.2 are in relatively fair agreement with the corresponding experimental measurements and zig-zag behaviors can be observed in all calculations (results based on other coefficients of friction are not shown); and (b) only the measured withdraw forces are reported here. The measured force for the terminal pulled out of the housing of the PCIE connector rises with respect to the increasing displacement, while the calculations display a similar trend. Nevertheless, the peak force based on the experiments emerges earlier than that based on the simulations, as shown in Figure 14a. For the SSD connector, there are obviously two measured peak forces (due to the design of terminal barbs) during the withdraw stroke as displayed in Figure 14b, although numerical results cannot replicate this phenomenon. The approaches commonly adopted in the industry without implementing the modified VBO model into the analysis (giving apparently low withdraw forces) are also illustrated in Figure 14a,b. Table 3 lists the retention forces of the PCIE and SSD connectors based on the measurements and simulations with various damage criteria. Calculated values based on the damage accumulation criterion, as expected, match well with measured ones. The other three damage criteria relatively underestimate or overestimate the retention force as demonstrated in the table.

## 4. Conclusions

Mechanical behaviors of the engineering polymers PA4T and LCP were experimentally and numerically examined in the current study. The modified VBO model, which was suitable for the rate-dependent semi-crystalline polymeric materials with four damage criteria, was coded as the user subroutine implemented into the finite element analysis software. Numerous material parameters of the modified VBO model can be evaluated by fitting the stress-stain relationships of the specimen under the uniaxial compressive loading conditions at two distinct strain rates based on the simulations with those based on the experiments. By utilizing the strength investigation of the latch of the DDR4 connector, simulations can reasonably capture the mechanical response and give fair estimations of crack orientation compared with the experimental observations. Trends of the withdraw force-displacement relationships of PCIE and SSD connectors based on the modified VBO model with the damage accumulation criterion and the corresponding experiments are relatively similar, and simulated retention forces agree well with the measured ones, while the approach commonly adopted in the industry yields substantial underestimations. Applications of the current numerical procedures to evaluate mechanical responses of other polymeric components/products of interest could be promising in the future.

## Figures and Tables

**Table 1:** Parameters adopted for the modified VBO model.

Description	Parameter	Parameter Value	
–	–	PA4T	LCP
Volume fraction of the crystalline phase	$\chi_{c}$	0.66	0.66
Young’s modulus	$E_{A}^{a}$	666.7 MPa	533.3 MPa
	$E_{A}^{c}$	1333.3 MPa	1066.7 MPa
Poisson’s ratio	$\nu_{A}^{a}=\nu_{A}^{c}$	0.4	0.4
Drag stress	$H^{a}=H^{c}$	40 MPa·s	25 MPa·s
Viscosity coefficient	$m^{a}$	2.8	2.6
	$m^{c}$	2.2	2.3
Tangent modulus	$E_{t}^{a}=E_{t}^{c}$	200 MPa	10 MPa
Material constants	$\beta^{a}=\beta^{c}$	0.45	0.70
	$\alpha^{a}=\alpha^{c}$	2	2.5
	$r^{a}=r^{c}$	200 MPa	60 MPa
Shape coefficients	$c_{1}^{a}$	80 MPa	180 MPa
	$c_{1}^{c}$	80 MPa	200 MPa
	$c_{2}^{a}$	200 MPa	230 MPa
	$c_{2}^{c}$	3000 MPa	3000 MPa
	$c_{3}^{a}=c_{3}^{c}$	25 s	15 s
	$c_{4}^{a}=c_{4}^{c}$	5	5
	$c_{5}^{a}=c_{5}^{c}$	3 MPa^−1^	3 MPa^−1^
Rubbery modulus	nkθ	2.1 MPa	2.1 MPa
Entanglement density	N	100	100

**Table 2:** Parameters adopted for various damage criteria.

Damage Criterion	Parameter	Parameter Value	
–	–	PA4T	LCP
Maximum principal strain	$\varepsilon_{1,\;cr}$	0.069	0.093
Equivalent viscoplastic strain	$\varepsilon_{D}^{vp}$	0.076	0.073
Damage accumulation	$p_{D}$	0.041	0.049
	Y	1.18	1.42
	$D_{cr}$	0.751	0.923
Craze-initiation	$f_{1}$	69.3 MPa	77.6 MPa
	$f_{2}$	532 MPa^2^	707 MPa^2^

**Table 3:** Comparisons of the retention forces of PCIE and SSD connectors based on the experiments and simulations with various damage criteria.

Damage Criterion	Retention Forces	
–	PCIE	SSD
Averaged experimental measurements	10.58 ± 1.15 N	1.78 ± 0.17 N
Maximum principal strain	11.6 N	0.77 N
Equivalent viscoplastic strain	11.9 N	1.27 N
Damage accumulation	10.3 N	1.53 N
Craze-initiation	10.2 N	1.19 N

## Data Availability

The data presented in this study are available on request from the corresponding author.
